# Novel Antibody-Based Proteins for Cancer Immunotherapy

**DOI:** 10.3390/cancers3033370

**Published:** 2011-08-19

**Authors:** Jaheli Fuenmayor, Ramon F. Montaño

**Affiliations:** Laboratorio de Patología Celular y Molecular, Centro de Medicina Experimental, Instituto Venezolano de Investigaciones Científicas. Caracas, 1020-A, Venezuela

**Keywords:** PMNs, NK-cells, T-cells, radioimmunoconjugates, immunocytokines, antibody fusion protein, immunotherapy, antibody-drug conjugates, combinatorial strategies

## Abstract

The relative success of monoclonal antibodies in cancer immunotherapy and the vast manipulation potential of recombinant antibody technology have encouraged the development of novel antibody-based antitumor proteins. Many insightful reagents have been produced, mainly guided by studies on the mechanisms of action associated with complete and durable remissions, results from experimental animal models, and our current knowledge of the human immune system. Strikingly, only a small percent of these new reagents has demonstrated clinical value. Tumor burden, immune evasion, physiological resemblance, and cell plasticity are among the challenges that cancer therapy faces, and a number of antibody-based proteins are already available to deal with many of them. Some of these novel reagents have been shown to specifically increase apoptosis/cell death of tumor cells, recruit and activate immune effectors, and reveal synergistic effects not previously envisioned. In this review, we look into different approaches that have been followed during the past few years to produce these biologics and analyze their relative success, mainly in terms of their clinical performance. The use of antibody-based antitumor proteins, in combination with standard or novel therapies, is showing significant improvements in objective responses, suggesting that these reagents will become important components of the antineoplastic protocols of the future.

## 1. Introduction

Passive immunotherapy with monoclonal antibodies (mAbs) represents one of the most relevant recent advances in cancer treatment. Initially envisioned as Paul Erlich's “magic bullets”, mAbs have proven to be much more than simple delivery vehicles for truly active compounds. There is now abundant literature reporting their association with objective responses against different kinds of malignancies in animal models, as well as in the clinic [1]. An inspection of the circumstances under which a therapeutic mAb induces a desirable antitumor effect should provide valuable information for the development of next generation Ab-based reagents with enhanced antitumor properties. Current clinical practice seems to be telling us that maximal effective and objective responses are achieved when various and different antitumor mechanisms are put into play. Two deductions can be drawn from the preceding ideas. The first one is that the “ideal” anticancer drug would be one that simultaneously combines several -as many as possible- mechanisms directed against the tumor. The second is that antibody technology can provide us with such new more versatile-antitumor molecules.

Novel mAb-based antitumor proteins have been designed based on results from studies on the mechanisms of action of current therapeutic mAbs, as well as from previous knowledge of the immune response and tumor biology. Accordingly, the general questions to answer with this kind of novel molecules are: have therapeutic mAbs' properties been improved, making them more potent cytotoxic/cytostatic agents? Has it been possible to add new properties to current and newly developed antitumor mAbs, turning them into more versatile antineoplastic weapons? In the present review, we look into different approaches that have been followed during the past few years, and analyze their relative success, mainly in terms of their clinical performance. An exhaustive record of all the new-generation therapeutic antibodies and their derivatives would be too long in the context of a concise review. Excellent extended reviews specific for each type of mAb-based reagent can be found in the literature and readers are referred to some of them [2-11]. Here, we have chosen recent examples to illustrate the relative success of each strategy. In addition, a more comprehensive list of therapeutic mAb-based antitumor proteins developed in the past five years is provided as supplementary material (Table S1).

We classified these new strategies according to their proposed mechanism of action. These mechanisms can be broadly grouped in two categories: those that target the cancer cell itself, *i.e.* the capacity to directly induce cytotoxicity or cytostaticity on cancer cells; and those that are elicited *in vivo*, *i.e.* the ability to trigger an effective antitumor response within the body. The relative importance of each type of mechanism on the final therapeutic benefit depends not only on their intensity, effectiveness and duration, but also on the particular characteristics of the malignant disease.

## 2. Improvement of Direct Toxicity to Cancer Cells

Cancer cells posses the ability to grow without control [12]. Therefore, the capacity to stop or delay their growth is a desirable feature of any antineoplastic therapy. Even though many of the available mAbs currently used in cancer therapy, such as rituximab (anti-CD20), trastuzumab (anti-HER2/*neu*), and cetuximab (anti-EGFR), have all been demonstrated to possess intrinsic antitumoral activity *in vitro* [6], significant cytotoxicity or cytostaticity is achieved at high concentrations. The observation that such activity depends on the capacity of the antibody to interfere with signaling cascades involved in tumor cell survival [6] have encouraged the development of a new generation of mAb-based therapeutic agents capable of inducing a higher degree of tumor cell toxicity. To fulfill this goal, several types of molecules have been conjugated to therapeutic antibodies and many of the initial drawbacks that precluded their use, such as immunogenicity, stability, potency, linker choice, homogeneity of the resulting products, *etc.* have been partially overcome [7]. Consequently, a significant number of mAbs conjugated to microtubule assembly or protein synthesis inhibitors, DNA-binding toxins, or radioisotopes are currently being tested in pre-clinical and clinical settings.

### 2.1. Antibody-drug Conjugates

Pre-clinical studies have clearly shown that incorporation of highly potent drugs (free drug potency in the order of 10^−9^ to 10^−11^ M) to therapeutic antineoplastic antibodies results in more effective reagents than using low potency drugs already approved for cancer therapy such as doxorubicin (free drug potency around 10^−7^ M) [7,13]. Auristatins and maytansinoids, for example, are highly active inhibitors of microtubule assembly/function [14]. The addition of one of these anti-mitotic agents, auristatin E, to a chimeric (human/mouse) IgG1 antibody directed against CD30 (brentuximab) led to the generation of brentuximab vedotin (or SGN-35), an antibody-drug conjugate (ADC) significantly more potent than the mAb alone for the treatment of refractory systemic anaplastic large cell lymphoma (ALCL). This molecule has been shown to induce cell cycle arrest and apoptosis *in vitro* [15]. During the 52nd Annual Meeting of the American Society of Hematology (December, 2010), results from a Phase II trial showed complete remission in 17 out of 30 ALCL patients and partial remission in nine others, for an objective response rate by investigator assessment of 87% [16]. An update of results from this study was given at the ASCO meeting in June, 2011 [17], confirming the encouraging performance and the moderate toxicity observed with this drug. Seattle Genetics (http://www.seagen.com/index.php) has announced in a press release that the US FDA accepted two Biologics License Applications (BLAs) for bretuximab vedotin, one for the treatment of relapsed or refractory ALCL patients, and the other for treatment of patients with Hodgkin lymphoma, in which the drug has also shown clinical benefit.

Trastuzumab emtansine is another ADC, which contains a maytansine derivative (DM1) conjugated to the FDA-approved trastuzumab (a humanized IgG1 antibody specific for the human epidermal growth factor receptor 2 HER2/*neu*). Barok *et al.* have recently shown that trastuzumab-DM1 inhibits tumor growth in a trastuzumab/lapaninib (a kinase inhibitor used in breast cancer therapy)-resistant mouse model through induction of apoptosis, ADCC and mitotic catastrophe [18]. The biological and clinical relevance of this last mechanism in the efficacy of trastuzumab-DM1 requires further investigation, but the acquisition of an additional anti-proliferative capacity may account for the reduction in tumor size observed in the trastuzumab-DM1-treated animals as compared to the trastuzumab-only group.

Preliminary data from a Phase II clinical trial in metastatic breast cancer patients compared the effect of trastuzumab emtansine with the standard treatment (trastuzumab plus docetaxel), and revealed a similar clinical benefit rate for both therapies: 55–57%. Importantly, the rate of clinically relevant adverse effects was significantly lower (37% vs 75%) in the trastuzumab emtansine group, including a reduction from 45% to 3% of alopecia cases [19]. These significant differences seem to imply that limiting the chemotherapeutic action by targeting a high-potency drug into the local tumor microenvironment renders a higher benefit to patients than the effect of a low-potency chemotherapeutic acting systemically. The lower general toxicity and its associated increase in life quality and better physical appearance in a patient population that are mainly females are important improvements of trastuzumab-DM1 that certainly deserve attention.

Gemtuzumab ozogamicin (Mylotarg) is another ADC that has been in the US market since its FDA approval in May 2000. Mylotarg is a humanized IgG4 anti-CD33 antibody conjugated to calicheamicin, a highly potent antibiotic that induces apoptosis in cancer cells by a DNA binding mechanism. Calicheamicin works at very low concentrations, allowing for its use at low doses *in vivo*. *In vitro*, gemtuzumab ozogamicin was 2000-fold more potent than the parental drug that preceded its development [20] and was the first ADC approved by the FDA licensed to treat recurrent acute myeloid leukemia (AML) in patients aged 60 and older who were not candidates for standard chemotherapy. The myeloid cell surface antigen CD33 represents an attractive target, as it is expressed on tumor cells from about 90% of AML patients. In spite of auspicious pre-clinical [20] and initial clinical [21] results, follow-up studies showed no additional benefits to AML patients, and an increased fatality rate in chemotherapy plus Mylotarg-treated patients when compared to standard treatments (5.7% vs. 1.4%). For these reasons, the product was voluntarily withdrawn from the US market in June 2010 (http://www.fda.gov/Safety/MedWatch/SafetyInformation/SafetyAlertsforHumanMedicalProducts/ucm216458.htm) [22]. Mylotarg is a heterogeneous formulation, containing approximately a 1:1 mixture of conjugated (one to eight calicheamicin moieties per IgG molecule, with an average of 4–6 moieties randomly linked to solvent exposed lysyl residues of the antibody) and unconjugated antibody [23]. The impact that this heterogeneity and the great potency of the drug could have had on the therapeutic performance of Mylotarg has not been addressed, but its clinical record suggests that further development and optimization is needed before a reevaluation of the therapeutic potential of this kind of conjugates is granted.

Inotuzumab ozogamicin (IO) is another humanized IgG4 antibody-calicheamicin conjugate; it recognizes the CD22 antigen. CD22 is a cell membrane protein expressed on the surface of mature B-lymphocytes and their malignant counterparts, but not on other cells of hematopoietic origin. CD22 is rapidly internalized after binding to inotuzumab. The addition of calicheamicin conferred the mAb a new apoptotic capacity *in vitro* [24]. IO is currently being tested in Phase I-II-III clinical trials for the treatment of CD22^+^ follicular B-cell Non Hodgkin's Lymphoma (NHL) (ClinicalTrials.gov identifier: NCT00562965) [25]. In a Japanese study, a small cohort of 13 relapsed or refractory CD22^+^ B-cell follicular NHL patients were treated with inotuzumab ozogamicin as a monotherapy, obtaining complete (54%) and partial (31%) responses, with the remaining 15% patients showing a stable disease. The overall response rate was 85% [26]. Results from a Phase III clinical trial in the US (ClinicalTrials.gov identifier: NCT00562965) should be available in August this year.

### 2.2. Immunotoxins

Pseudomonas exotoxin A (PE) acts as a protein synthesis inhibitor [27]. Due to its great cytotoxicity and bacterial origin, PE needs to be modified to avoid non-specific toxicity and reduce its immunogenicity when used in therapy [28]. An immunotoxin (IT) combining a stabilized and affinity-improved Fragment variable (Fv) of an antibody specific for CD22 (RFB4) with a truncated version of PE (PE38; which is devoid of the PE N-terminal cell binding domain) has been engineered [29-33]. The molecule, named HA22 or CAT-8015, displays improved antitumor cytotoxic activity and is being tested in Phase I/II clinical trials for the treatment of chronic lymphocytic leukemia (CLL), hairy cell leukemia (HCL), and NHL. Results from a Phase I study, presented at the 2010 Annual Meeting of the American Society of Clinical Oncology, showed a low HA22-related general toxicity and an overall response rate of 79% in the group of HCL patients, including 12 out of 28 (43%) who achieved complete remission [34].

HA22-8X and HA22-LR-6X are more recent versions of HA22. They have been further modified by deletion of PE38 B-cell epitopes [35,36]. In experimental animal models, HA22-8X and HA22-LR-6X showed marginal PE-related immunogenicity and preserved the antitumor cytotoxic properties of parental HA22. This protein engineering approach may be applied to the generation of similar ITs that target solid tumor-related antigens. Such HA22-8X/HA22-LR-6X-like ITs with marginal immunogenicity/antigenicity should have a major therapeutic impact on the treatment of cancer patients with non-hematologic malignancies, since these patients generally have normal immune functions and, consequently, a high risk of developing neutralizing anti-immunotoxin antibodies. HA22, HA22-8X, and HA22-LR-6X are also clear examples that illustrate the formidable obstacles faced when developing Ab-based drugs with high therapeutic potential. At the same time, these molecules represent excellent testimonies of the usefulness of recombinant antibody technology in cancer immunotherapy.

### 2.3. Radioimmunoconjugates

^90^Yttrium-ibritumomab tiuxetan (^90^Y-IT or Zevalin) and ^131^Iodine-tositumomab (^131^I-T or Bexxar) are two radioisotope-conjugated murine monoclonal antibodies approved by the FDA in 2002 and 2003, respectively. Both radioimmunoconjugates (RICs) target CD20, the same tumor antigen as ofatumumab and rituximab (ibritumomab is actually the murine IgG1/κ mAb from which chimeric rituximab was made of; tositumomab is a murine IgG2a/λ). Besides maintaining the tumoricidal properties of the unconjugated antibodies (which include apoptosis, complement-dependent cytotoxicity, and Ab-dependent cell-mediated cytotoxicity), they deliver ionizing β-radiation to target cells and cells in the vicinity, inducing cell death by “a cross-fire” mechanism. They are indicated for the treatment of patients suffering from CD20^+^, low-grade or follicular B-cell NHL who have relapsed or that do not respond to rituximab [37].

When initially compared in a large randomized study, ^90^Y-IT resulted more effective than rituximab (80% versus 56% in overall response rate; and 30% versus 16% in complete response rate) for the treatment of patients with relapsed or refractory low grade or transformed B-cell NHL [38]. Subsequent clinical studies suggested that ^90^Y-IT performs equally better in older patients [39], and it is also safe and effective as second-line therapy for patients with relapsed disease [40]. Several clinical studies have documented a similar performance for ^131^I-T [41,42].

More recently and based on results from Phase II/III clinical trials [43-46] regulatory organisms in USA and EU (FDA and EMEA, respectively) licensed the use of ^90^Y-IT as a first-line consolidation therapy in follicular NHL patients who show a favorable response to induction with a combined chemo/immunotherapy protocol (http://www.ema.europa.eu/docs/en_GB/document_library/EPAR_-_Product_Information/human/000547/WC500049469.pdf) [47,48].

In summary, boosting antineoplastic mAbs with cytotoxic moieties has not been an easy task. From conjugation to immunogenicity to adverse side effects due to systemic toxicity, every step has necessitated thoughtful insights to attain clinical-grade reagents. Indeed, only a handful of the many IT, ADC or RIC molecules tested in preclinical studies have reached clinical development. Nevertheless, all these efforts certainly improved the performance of the original mAbs, and the capacity to increase the cell death induction potential appears to be an important feature of new-generation mAb-based antineoplastic proteins. This is a dynamic research area and many new molecules are being developed using the positive features of the ones that have made it to the clinical trials but including new features that wait for testing. Numerous examples can be found in the literature, including mAbs directed to various tumor targets conjugated to the same or similar drugs to the ones already mentioned [3,49]. Additionally, the discovery of anti-cancer properties in molecules such as anti-microbial and cationic peptides [50,51], and the use of proteins of human origin such as pro-apoptotic Bax, Bak; granzyme B, TRAIL, FasL, C5a and RNAse [28,52,53] may pave the way for a future generation of antibody-based toxic proteins with clinical applications in cancer therapy.

## 3. Targeting Tumor-Host Interaction

### 3.1. Targeting Angiogenesis

An important event during tumor progression is the neovascularization of the malignant tissue. This process of angiogenesis is necessary to provide tumor cells with oxygen and nutrients, and critically depends on the interaction of vascular endothelial growth factors (VEGFs) with receptors (VEGFRs) found on the surface of endothelial cells [54]. Aflibercept (VEGF Trap or ZALTRAP™) is a genetically engineered soluble fusion protein that combines the extracellular Ig domains 2 of VEGFR1 and 3 of VEGFR2, with the Fragment, crystallizable (Fc) portion of the human IgG1. Aflibercept acts as a decoy receptor molecule that traps VEGFs, impeding the interaction with their receptors at the surface of vascular endothelial cells [55]. Phase I/II clinical trials established that no anti-aflibercept antibody response is produced in humans. The two major side effects, associated with the use of aflibercept in patients suffering from adenocarcinoma of the lung, ovarian cancer, glioblastoma, and melanoma, were hypertension and proteinuria [56-59]. Randomized Phase III clinical trials testing the use of aflibercept in combination with chemotherapy for the treatment of colorectal, non-small-cell lung, prostate, and pancreatic cancer are currently underway [60]. In a recent press release (http://en.sanofi.com/binaries/20110426_VELOUR_en_tcm28-31928.pdf) Sanofi-Aventis and Regeneron commented on the first results from the VELOUR trial (a study in metastatic colorectal cancer patients that is comparing a combined regimen of chemotherapy plus aflibercept versus chemotherapy alone) indicating “exciting positive findings…… will be presented at an upcoming medical meeting”. In addition, they disclosed their plan to submit regulatory applications for marketing approval to the US FDA and the European Medicines Agency later this year.

Amgen is developing a similar antibody Fc fusion protein, AMG 386. AMG 386 is a “peptibody” composed of an angiopoietin-binding peptide fused to the Fc portion of human IgG1. AMG 386 inhibits the interactions of angiopoietin 1 (Ang-1) and Ang-2 with their receptor, Tie2, thereby affecting angiogenesis. After recent results from Phase I and II clinical trials, where no anti-AMG 386 antibody response could be documented and drug safety was corroborated, Phase III studies started at the end of 2010 [60].

### 3.2. Triggering an Effective Antitumor Response

#### 3.2.1. Immunocytokines

Elucidation of the different mechanisms of action through which therapeutic mAbs exert their antitumoral effects has highlighted the importance of the immune system for the success of this kind of therapy. Since then, many immunologically active molecules have been combined with mAbs in various formats. One of the most important group of such molecules are cytokines. Some cytokines, including Interleukin (IL)-2, Granulocyte-Macrophage Colony-Stimulating Factor (GM-CSF), Tumor Necrosis Factor alpha (TNF-α), and IL-12, have been demonstrated to induce desirable clinical responses in cancer patients [61]. Their main drawback is the severe systemic toxicity associated with the high blood concentrations of cytokine needed to obtain an antitumoral effect. This gave rise to a generation of cytokine-antibody fusion proteins (or immunocytokines) targeting cytokines directly to the tumor, seeking to take advantage of the benefits of cytokines in cancer therapy while reducing their undesirable systemic side effects [4]. Currently, several immunocytokines are in Phase I and II clinical trials, representing the group of antibody fusion proteins that are closer to FDA approval. Table 1 shows the immunocytokines for which clinical trials are currently under way; a more detailed description of recent clinical trial results for some of them follows.

During cancer progression the alternatively spliced extra-domain B (ED-B) of fibronectin is strongly expressed in neo-vascular and stromal structures, but it is rarely expressed on normal cells, which makes it a suitable antigen for solid tumor therapies. As seen on Table 1, ED-B is the target of several strategies currently being tested in patients. One therapeutic strategy combines a humanized IgG1 antibody that recognizes ED-B (huBC1) with IL-12. huBC1-huIL12 (or AS1409) is a hexameric fusion protein (300 kDa) that has been evaluated in a Phase I study in malignant melanoma and renal cell carcinoma patients. The observed toxicities were manageable and predictable at the maximum tolerated dose (15 μg/kg) and they were less prominent than those observed with IL-12 as a single agent. Of 11 patients with malignant melanoma in the trial, one achieved a sustained partial response and another achieved a 29% reduction in measurable target lesions' sizes. Five other patients showed stable disease [62].

L19-IL2 consists of human interleukin-2 (IL-2) fused to the single chain Fv (scFv) antibody fragment L19. L19 also recognizes fibronectin ED-B. In a Phase I/II trial, application of this immunocytokine to advanced renal cell carcinoma patients produced tolerable and reversible toxicities, along with the induction of stable disease in 17 out of 33 patients, 15 of whom had metastatic disease. Two patients remained progression-free for at least 27.5 and 30.5 months after termination of the treatment, resulting in a progression-free period without toxicity. This is an important benefit of L19-IL2, since other protocols used in these patients require sustained therapies to prevent progression, which involve associated toxicities [63].

Hu14.18-IL2 combines an antibody against the disialoganglioside GD2, a carbohydrate antigen found on melanomas, neuroblastomas and some sarcomas, with IL-2. In a recent Phase II study, hu14.18-IL2 induced complete regression of neuroblastoma lesions in five out of 23 patients with minimal disease evaluable only by [^123^I] metaiodo benzylguanidine (MIBG) scintigraphy and/or bone marrow (BM) histology. Thirteen other patients with bulky disease did not respond to therapy [64]. Tumor biopsies of patients with metastatic melanoma treated with hu14.18-IL2 evidenced T-cell, but not NK cell infiltration after treatment [65].

Many of the mechanisms through which immunocytokines exert their antitumoral effects have been studied in animal models and include: induction of IFNγ secretion, T-cell and NK-cell activation, tumor cell apoptosis, ADCC enhancement, increased polymorphonuclear adhesion and degranulation, *etc.* [4]. Interestingly, some immunocytokines have been shown to induce an immune response capable of providing protection against tumor cell challenges in mice [66].

#### 3.2.2. Harnessing T-cells

Currently, lymphocyte infiltration is considered one of the best indicators of prognosis for certain tumors [67,68]. For years, induction of specific T-cell responses against tumor cells has been one of the most pursued objectives in cancer immunotherapy. Besides fusing mAbs to cytokines, T-cell infiltration is also induced by combining therapeutic mAbs with T-cell-stimulating molecules, such as superantigens. This is done by directly activating antigen-presenting cells using fusion proteins, or by designing bispecific mAbs directed to both T-cell and tumor cell markers.

Naptumomab estafenatox, for example, is a recombinant fusion protein consisting of a mutated variant of the staphylococcal Superantigen Enterotoxin E (SEA/E-120) linked to the fragment, antigen binding (Fab) of a monoclonal antibody recognizing the oncofetal trophoblast glycoprotein 5T4. This antibody fusion protein showed evidence of antitumoral and immunological activity in Phase I studies in patients with non-small-cell lung cancer and renal cell cancer. Thirty six percent of the patients showed stable disease at day 56 (25% of the non-small-cell lung cancer patients and 64% renal patients) when used as a single agent, and 38% when used in combination with docetaxel. Two patients (15%) from the combination group showed partial responses that continued after 30 months. Immunohistochemistry of biopsy samples of two patients showed T-cell infiltration after treatment [69]. Another study evaluated naptumomab estafenatox activity in combination with IFNα for the treatment of advanced renal carcinoma. Results from this study are expected to be disclosed later this year [60].

A different approach takes advantage of antigen presenting cell (APC) activation to achieve lymphocytic stimulation. Lymphocyte Activation Gene-3 (LAG-3) is a MHC class II ligand related to CD4. A soluble form of LAG-3, termed LAG3Ig, was generated by genetically fusing DNA encoding the extracellular domain of human LAG-3 and the constant region of human Ig γ1 heavy chain spanning the hinge, C_H_2, and C_H_3 domains [70]. Binding of LAG3Ig to MHC class II molecules has been reported to act as an adjuvant, driving human immune responses toward a T-helper type 1 (Th1) phenotype [71], and to induce the activation of a large range of human effector cytotoxic cells [72]. In a Phase I clinical trial in metastatic breast carcinoma patients, the addition of LAG3Ig to the standard treatment (paclitaxel), did not induce clinically significant adverse effects, and no anti-LAG3Ig antibodies were detected (which is somewhat expected due to its human origin). Most importantly, only three out of 30 patients had disease progression in six months and the objective response rate increased from 25% in the historical control group to 50% in the trial group [73]. Even though these clinical results are encouraging and were accompanied with increases on the activation state and absolute numbers of circulating monocytes, NK and CD8^+^ T-cells, a recent observation showing that MHC class II-positive tumors, in particular malignant melanoma, can use the MHC class II-LAG3 interaction to become apoptosis-resistant [74] may limit the use of LAG3Ig as an antitumor therapeutic to MHC class II-negative tumors.

A novel kind of antibody construct, called BiTE (for “Bispecific T-cell Engager”), has shown promising results in clinical trials. BiTEs are antibody constructs that contain two ScFv of different specificities, one directed to the T-cell and the other one to the cancer cell. This maneuver brings together both cells, redirecting the T-cell lytic activity irrespectively of the T-cell specificity. Blinatumomab, a construct that binds CD19 on B-cells and CD3 on T-cells, was the first BiTE tested in patients. The first clinical trial showed an overall response rate of 82% as monotherapy in B-cell NHL. The most relevant adverse effects at the onset of the treatment were reversible central nervous system events that led to discontinuation of therapy in 14 of 62 patients. However, in 21 adult B-cell precursor acute lymphoblastic leukemia patients with signs of minimal residual disease (MRD), Blinatumomab induced the conversion from MRD-positive to MRD-negative in 16 patients. This result is of major importance since MRD in B-cell precursor acute lymphoblastic leukemia is a poor prognosis marker for which no satisfactory treatment options are available [75].

Another BiTE antibody construct, directed against the epithelial cell adhesion molecule (EpCAM) and CD3, was tested in a Phase I study showing low toxicity and some biological activity in patients with solid tumors. Disease stabilization was achieved in seven out of 19 patients and CD8^+^ T-cell counts increased in five of them [75,76].

#### 3.2.3. Harnessing Innate Immunity Effector Cells

The notion of involvement of cells from the innate immunity in the anti-cancer response mainly comes from studies on the mechanisms of action associated with successful responses in patients. Complement-dependent cytotoxicity (CDC) and more prominently antibody-dependent cellular cytotoxocity (ADCC) have been implicated in the success of FDA-approved therapeutic antibodies such as rituximab and trastuzumab [77,78]. Notably, patients with partial and complete responses to trastuzumab showed an increased number of total leukocytes in their biopsies, whereas the number of infiltrating lymphocytes did not differed between responders and non-responders. Also, these responding patients showed higher ADCC activity *in vitro* [79]. Since ADCC is accomplished by cells of the innate immunity, an increased interest in these immune cell populations is arising.

##### 3.2.3.1. NK cells

NK cells have been considered one of the main ADCC effectors in cancer immunotherapy [80]. They show mAb-mediated cytotoxic activity against tumor cell lines *in vitro* [81] and express CD16a on their surface, an important immunoglobulin receptor with polymorphic variants that has been associated with clinical responses to rituximab, trastuzumab and cetuximab [82-84]. Even though NK cells possess an undisputable cytotoxic potential, the NK cell populations isolated from cancer patients show impaired antitumor functions [85], low cell counts and decreased cytotoxicity *in vitro* [11]. Restoration of NK cell's antitumoral properties and enhancement of NK-mediated ADCC have been the focus of some immunotherapeutic approaches including stimulation with mAbs fused to cytokines such as IL-2, IL-12 and GM-CSF [4].

In a recent approach, Kellner *et al.* increased NK-mediated cytotoxicity against malignant cells isolated from patients by improving the avidity for CD19 of a CD19/CD16 bispecific Fab-based protein [86]. They compared a bibody construct (one ScFv against CD16 and one Fab fragment against CD19) with a tribody construct (one ScFv against CD16, one ScFv against CD19, and one Fab fragment against CD19) in terms of binding and cytotoxicity. Having a 3-fold higher avidity for CD19 and equal affinity for CD16 compared to the bibody format, the tribody mediated the lysis of a higher percent of target cells by NKs isolated from healthy donors.

Cell number is also an important concern in NK-based cancer immunotherapy, because these cells are present in relatively low numbers in peripheral blood. Therefore, expansion and activation of NK cells has been pursued through several strategies, including incubation with cytokines such as IL-2 and IL-15 [11,87]. Recently, Wu *et al.* expanded NK cells isolated from healthy donors using an Ab-based chimeric molecule consisting of the human IL-15 receptor, IL-15Rα, fused to the Fc portion of IgG1. NK cells expanded in such manner lysed a significantly higher number of tumor cells (K562) in the presence of IL-2 than NK cells stimulated with IL-2 or IL-15 alone [88].

NK cell ability to target cancer cells not only depends on MHC class I expression on the tumor cell, but also on a set of surface receptors on the NK cell named the natural killer cytotoxic receptors (NCR). One of these molecules, the NKp30 receptor, identifies cancer cells through an unknown ligand. This property has been translated into a therapeutic tool by developing a fusion between the extracellular domain of NKp30 and the constant region of a human IgG1. Treatment of tumor xenografts in nude mice with this antibody fusion protein results in complete remissions in half of the animals and partial responses in another 25%. *In vitro* ADCC activity induced by this fusion protein was mediated by activated peritoneal macrophages [89], but no other cell types were tested in these ADCC assays.

##### 3.2.3.2. PMNs

Even though NK cells and macrophages have been identified as important populations involved in ADCC of tumor cells, most of these studies have been performed using peripheral blood mononuclear cells obtained from human subjects. The polymorphonuclear (PMN) population is normally removed from these samples during the isolation procedure. Additionally, high neutrophil : lymphocyte ratios in peripheral blood of cancer patients have been associated with poor prognosis, an observation that may depict PMNs as cells with little therapeutic potential [90,91]

Myeloid-derived suppressor cells (MDSC) are a subset of a heterogeneous population that infiltrate tumors and make them poorly immunogenic [92]. In mice, MDSCs consist of cell subsets in intermediate myeloid stage of development that possess a high immunoregulatory potential. These cells can differentiate toward a monocytic or granulocytic lineage depending on the tumor microenvironment [93]. In cancer patients, PMNs isolated from the MDSC fraction of peripheral blood show an immature phenotype with impaired migratory properties and T-cell suppressive capacities [94].

On the other hand, fully mature, active PMNs are the most cytotoxic and numerous of all leukocytes and evidence from experimental models support their possible use in cancer immunotherapy. For instance, a chemoimmunotherapeutic protocol that eradicates colon cancer tumors in mice induces the disappearance of MDSCs and the concomitant appearance of inflammatory myeloid cells within the tumor. Depletion of this emerging inflammatory myeloid population by treatment with a specific antibody (anti-Gr1 mAb) not only abolished the anti-tumor response of the therapy, but also abrogated the infiltration of effector T-cells [95].

Additionally, the importance of PMNs in the activity of rituximab was evidenced using a B-cell lymphoma SCID mouse model, in which depletion of neutrophils dramatically reduced the efficacy of the treatment, even in the presence of intact NK cells [96].

The way PMNs can exert an antineoplastic response is not completely understood, but chemoattraction of active PMNs to the tumor site is one important requirement. Studies on a mouse colony that show spontaneous regression of tumors and cancer resistance reveal that tumor regression requires both the infiltration of tumors with leukocytes, particularly PMNs, and the recognition of some undetermined surface markers on tumor cells [97].

In addition to what has been observed in animal models, *in vitro* studies show that PMNs can induce Ab-mediated apoptosis in a human breast cancer cell line (SK-BR 3) [78]. In the clinic, administration of granulocyte-colony stimulating factor (G-CSF) during radiotherapy significantly increased the overall survival of patients with squamous head and neck cancer. G-CSF activates and mobilizes PMNs, suggesting that this cell population might have been partly responsible for the observed improved survival [98]. With this in mind, some recent immunotherapeutic approaches focus on the exploitation of PMN's antitumoral properties, taking advantage of their high numbers and lack of specificity [99]. In a clinical trial currently underway, granulocytes from healthy donors are mobilized with dexamethasone and G-CSF, collected via apheresis, and then infused to cancer patients (Clinicaltrial.gov identification number: NCT00900497).

Initial attempts to induce PMN-mediated ADCC in cancer patients using a bispecific antibody (MDX-H210) directed toward HER2/*neu* and the Fc gamma receptor I (FcγRI) were not as encouraging as expected [100]. Either alone or combined with GM-CSF, G-CSF or IFN-γ, no significant clinical responses were observed. A plausible explanation holds that recruitment of PMNs through FcγRI selects for immature PMNs with no antitumoral capabilities [5]. In the mean time, new Ab-based anti-tumor proteins targeting receptors other than FcγRI on PMNs have been developed. For instance, a recombinant single chain Fv fragment specific for HLA class II, on the tumor cell, and for the IgA Fc receptor (FcαR or CD89) on PMNs [(FcαRI × HLA class II) bsscFv] has recently been engineered and tested in the context of B-cell malignancies. CD89 is preferentially expressed on mature neutrophils and it stands as the most potent neutrophil FcR for ADCC [101]. (FcαRI × HLA class II) bsscFv effectively mediates the killing of B-cell lymphoma cell lines and human primary tumor cells obtained from patients with B-cell malignancies that are resistant to lysis by other anti-HLA class II-specific antibodies *in vitro*. As expected, PMNs were identified as the relevant effector population [102].

Another Ab-based protein that stimulates PMNs through a receptor other than FcγRI is anti-HER2/*neu*-(C5a) [52]. Anti-HER2/*neu*-(C5a) is a human IgG3 molecule specific for HER2/*neu* fused to the C5a fragment derived from the component C5 of the complement system. *In vitro*, this antibody fusion protein attracts human PMNs, binds and signals PMNs through the C5a receptor (C5aR), and induces the expression of Mac-1. Mac-1 has been shown to be essential for PMN-mediated ADCC, promoting binding to tumor cells and allowing formation of intercellular synapses [103]. Anti-HER2/*neu*-(C5a) also possesses the capacity to induce the death of HER2-expressing tumor cell lines, both in the presence or absence of effector cells, through some yet unknown mechanisms. The multiple features of anti-HER2/*neu*-(C5a) might contribute to the targeting of tumor cells through a multi-prong effector approach, *i.e.* direct cytotoxicity, chemoattraction of effector populations and stimulation of an immunological response.

From the clinical results observed when activating an immune response through Ab-based antitumor therapeutic, it becomes apparent that the induction of this type of response is important but not sufficient to achieve complete and durable cancer remissions. In several studies the most common clinical response observed is stable disease. It is possible that the circumstances that have warranted complete responses in experimental animals are not achievable in the main cancer patient populations. Often, cancer patients are older and/or heavily pre-treated people in which the immune potential is limited. Still, activation of an immune response should be a major component of antineoplastic therapies, due to the important role that immunity has on cancer development [104]. Perhaps, the simultaneous targeting of other tumor-promoting components at the time of immune stimulation might help solve these issues to attain more effective and robust antitumoral reactions. Additionally, controlling the influence of relevant immunoregulatory cells such as Tregs [105], MDSCs, and B-cells [106] could also help increase objective responses through immune activation.

## 4. Multiple Effects and Combinatorial Approaches

Since the War on Cancer started 40 years ago, cancer therapy has certainly improved. However, complete remissions as those achieved for many bacterial infection diseases are rarely observed. From the experience gained through infection disease research, one could extrapolate some of the parameters that need to be controlled in order to succeed. Tumor burden, immune evasion, physiological resemblance, and cell plasticity are among the features that complicate the picture and promote cancer progression in spite of research and clinical efforts. Traditional approaches such as surgery, chemotherapy and radiotherapy have focused on controlling tumor burden. Immunotherapeutic approaches try to deal with immune unresponsiveness and evasion. Genetic and biochemical studies seek for identification of important physiological differences that can serve as targets. Approaches involving the disruption of structural targets such as cell or mitochondrial membranes (which are difficult to mutate) could help overcome the tremendous cellular plasticity of cancer cells.

No antineoplastic antibody or antibody-based therapy has rendered reproducible and definite complete remissions and it is hard to imagine a single agent that could deal with all tumor strategies simultaneously. Good approaches are becoming more common with the development of different therapeutic agents that contribute to the resolution of these issues. Combination of these approaches can contribute to avoid the occurrence of relapses and the development of resistance mechanisms by targeting more than one tumor cell population simultaneously. This is known as the 10^−5^ × 10^−5^ = 10^−10^ rule, in which 10^−5^ refers to the approximate frequency of appearance of new mutants in a cancer cell population. Thus, by targeting tumor cells through multiple mechanisms, the probability of a new mutant escaping from the therapy-induced death will be significantly reduced [107]. Additionally, combination therapies may show synergistic effects not previously envisioned.

Several small molecules are being tested in combination with mAbs for cancer immunotherapy. Combination with protein-kinase inhibitors is currently under study in many clinical trials testing their possible additive and/or synergistic effects. Other molecules, such as small interfering RNA (siRNA), peptides, carbohydrate moieties and Ab-based constructs have been used to boost additional antineoplastic feature of mAbs. Complement-dependent cytotoxicity (CDC) and Complement Receptor 3 (CR3; CD11b/CD18)-dependent cellular cytotoxicity (CR3-DCC) are important mechanisms through which tumor cells can be eliminated. However, many tumor cells overexpress membrane-bound complement regulators (mCRegs), which inhibit the biological effects of complement activation induced by therapeutic mAbs [108]. To overcome this drawback, siRNAs that silence mCReg genes were developed, but this approach still lacks a safe and reliable mode of systemic delivery [109]. Alternatively, transfection of tumor cell lines having altered protein-kinase activity with a plasmid encoding a peptide called REST68 regulates the expression of CD59 (a mCReg of the terminal phase of the complement cascade) and increases CDC, apoptosis, and ADCC [110]. Even more, activity of antitumor mAbs in a mouse model can be enhanced by β-glucan administration, which primes CR3 on PMNs [111]. This receptor, also known as Mac-1, mediates binding to complement fragments iC3b deposited on tumor cells, promoting cell-to-cell contact and triggering cytotoxicity [103]. Finally, combination of antineoplastic mAbs with a Complement Receptor 2 (CR2)-Ab construct (CR2-Fc) significantly enhanced CDC of a human prostate cell line and improved the long-term survival of nude mice challenged with tumor cells [112]. CR2 also binds C3 fragments deposited on the surface of tumor cells. These evidences support the notion of complement activation as an exploitable additional antineoplastic mechanism induced by mAbs.

In animal models, complete eradication of, and protection from, human B-cell lymphoma xenografts were achieved by combining rituximab with L19-IL2. Staining of tumor sections with NK-and macrophage-specific antibodies revealed the presence of these populations among the infiltrating cells [113]. Furthermore, administration of IFN-α or docetaxel along with a superantigen-antibody fusion protein (C215Fab-SEA) prolonged the median survival time and reduced the number of lung metastasis in melanoma bearing C57Bl/6 mice [114,115]. In the clinic, the combination of different strategies has also proved more effective than single agents, particularly in terms of duration of the responses. For example, a Phase II study on the efficacy and safety of a combination chemotherapy (cyclophosphamide, vincritine and prednisone) followed by tositumomab and ^131^I-tositumomab was evaluated in patients with low-grade follicular lymphoma. For 30 patients evaluated, the response rate after completion of the therapy was 100% with 93% achieving complete remission. Five-year progression-free and overall survival rates were 56% and 83%, respectively [116]. Comparatively, a similar study using ^131^I-tositumomab as single agent produced a response rate of 83% and complete remission in 46% of 28 low-grade Non-Hodgkin's lymphoma patients. The median progression-free survival for responders was also much lower (12.3 months) [117].

The exact mechanisms for the improvements observed when using combination therapies have not been characterized but a recent article using experimental animals might give some important clues. Ramakrishnan *et al.* observed a synergistic effect of the combination of chemotherapy with cytotoxic T-cell transfer immunotherapy. The proposed mechanism involves the up-regulation of cation independent-mannose 6 phosphate receptor (CI-MPR), a 300 kDa transmembrane protein which over-expression is known to induce cell growth inhibition, by three different chemotherapeutic agents (paclitaxel, cisplatin or doxorubicin) [118]. This up-regulation potentiated cytotoxic T-cell killing by making tumor cells more permeable to granzyme B [119]. This hypothesis needs to be validated in patients, but some unexpected clinical benefits observed in patients having received cancer vaccines prior to chemotherapy support it [120]. Improvements in overall response rates suggest that combination of new agents with standard and novel therapies will probably become first line protocols for cancer therapy in the near future.

## 5. Concluding Remarks

The number of antibody-based antitumor proteins generated during the past years is almost countless. Their path to patients is long and costly [121]. Thus, a thorough analysis of the circumstances that lead to success and those that do not is mandatory. Such an analysis seems like an epic task even for those mAbs that have been widely used in the clinic, such as rituximab [122]. Even then, the design of new therapeutic Ab-based proteins on the basis of such scientific observations have not been as successful as expected [123], and curiously enough, the enhancement of some mechanisms of action have negatively affected the contributions of some others [122].

This reality reflects the complexity of the problem and demands for more individualized solutions. From our perspective, the ideal protocol should combine agents that: (1) promote direct and specific toxicity to tumor cells and/or cells in their microenvironment; (2) attract and activate a significant number of cytotoxic effector cells into the tumor microenvironment; either from innate immunity, adaptive immunity or both; (3) specifically inhibit the activity of relevant regulatory cell populations such as Tregs, MDSC, or B-cells; (4) cause the lowest possible toxicity to normal cell and tissues, and if possible; (5) interact synergistically. This may be easier said than done, but fine-tuning of the many options already in scope will certainly lead us to the goal of complete and durable remissions.

## Figures and Tables

**Table 1.** Immunocytokines currently on clinical trials.

**Antibody fusion protein**	**Condition**	**Intervention**	**Phase**	**Clinicaltrial.gov**
HuBC1-IL12 (Anti-EDB fused to IL-12)	Metastatic melanoma and renal cell carcinoma	Alone	I	NCT00625768
L19-IL2 (Anti-EDB fused to IL-2)	Advanced solid tumors	Alone	I /II	NCT01058538
	Malignant melanoma	Alone	II	NCT01253096
	Metastatic melanoma	Dacarbazine	II	NCT01055522
	Advanced pancreatic cancer	Gemcitabine	I/II	NCT01198522
L19-TNFα (Anti-EDB fused to TNFα)	Colorectal cancer	Alone	I/II	NCT01253837
	Melanoma	Mephalan	I	NCT01213732
F16-IL2 (Anti-tenascin C fused to IL-2)	Advanced solid tumor	Doxorubicin	I/II	NCT01131364
	breast cancer	Paclitaxel	I/II	NCT01134250
Hu14.18-IL2 (Anti-GD2 fused to IL-2)	Melanoma	Alone	II	NCT00109863
	Melanoma	Alone	II	NCT00590824
	Neuroblastoma	Alone	II	NCT00082758
	Neuroblastoma	GM-CSF and Isotretinoin	II	NCT01334515
DI-Leu16-IL2 (de-immunized anti-CD20 fused to IL-2)	Lymphoma	Alone	I	NCT00720135

## Supplementary Files

Supplementary File 1 PDF-Document (PDF, 191 KB)
