# Supplementary material for: Novel Antibody-Based Proteins for Cancer Immunotherapy
Source: Cancers (Basel). 2011 Aug 19;3(3):3370–93. doi: 10.3390/cancers3033370 (PMC3759200; doi:10.3390/cancers3033370)
Supplement: Supplementary File 1 — PDF-Document (PDF, 191 KB) [file cancers-03-03370-s001.pdf]

Supplementary file

Table S1. Representative examples of Ab-based antitumor proteins for cancer immunotherapy recently reported in the literature.

TARGET Ag	TARGET TUMOR	Ab FORMAT	FUSED PROTEIN OR CONJUGATED MOIETY	DRUG NAME	Ab PROPERTY/ INTRINSIC ACTIVITY	ADDED PROPERTY	RELEVANT COMMENT	Ref.	a	b	c	d	e	f
5T4	Pancreatic, non-small-cell lung (NSCLC) and renal cancer	Murine Fab	Mutated Staphylococcal Enterotoxin A	Naptumomab estafenatox	Tumor targeting	Superantigen-mediated T-cell cytotoxocity	Stable disease in 36% of NSCLC patients	[1]						
Carbonic anhidrase IX	Non-metastatic renal cell carcinoma	Chimeric (Mouse x Human) IgG1 (C_H2-C_H3 truncated)	Human TNF	c(G250)-TNF	Tumor targeting	Improved survival	Synergy observed when administered in combination w/IFN□	[2]						
CCR4	Adult T-cell Leukemia/ Lymphoma	Defucosylated humanized IgG1	none	Mogamulizumab (KW-0761)	Tumor targeting	Increased ADCC	NK dependent	[3]						
CD4	Cutaneous T-cell lymphoma	Human IgG1	none	Zanolimumab	Receptor downmodulation, alteration of signal transduction	ADCC	Phase III trial results due on Feb 2011	[4]						
CD20	Non-Hodgkin's Lymphoma (NHL)	Murine IgG2a	Isotope 131Iodine	131I-Tositumomab	Tumor targeting	Radiation delivery, bystander effect	93% complete remission, combined w/chemotherapy	[5]						2003

Table S1. *Cont*.

TARGET Ag	TARGET TUMOR	Ab FORMAT	FUSED PROTEIN OR CONJUGATED MOIETY	DRUG NAME	Ab PROPERTY/ INTRINSIC ACTIVITY	ADDED PROPERTY	RELEVANT COMMENT	Ref.	a	b	c	d	e	f
CD20 (Cont.)	NHL	Murine IgG1	Isotope ^{90}Yttrium	^{90}Y-Ibritumomab tiuxetan	Tumor targeting	Radiation delivery, bystander effect	72% complete remissions after Rituximab with chemotherapy	[6]						2002
	Lymphoma model		41-BBL (CD137L)	4-1BBL/anti-CD20	Tumor targeting	Increased T-cell cytotoxicity	Combined w/anti-CD3-CD20 diabody, leads to tumor inhibition	[7]						
	Lymphoma model	Recombinant deimmunized murine V Human IgG1	IL-2	DI-Leu16(IL-2)	Tumor targeting	Enhanced ADCC	Activity partially dependent on FcR binding	[8]						
	Lymphoma model	Chimeric (Mouse x Human) IgG3	IFN-α	Anti-CD20-IFN-α	Tumor targeting	Increased apoptosis, but not ADCC	Eradication of tumor xenografts required IFN receptor expression	[9]						
CD21	Prostate model	Human IgG1 Fc	CR2	CR2-Fc	Complement activator	Enhanced CDC and ADCC	In combination w/anti-MUC1 Ab	[10]						
CD22	Hairy cell leukemia	dsFv	Pseudomonas Exotoxin A	CAT-8015, HA22	Tumor targeting	Transcription inhibition	Complete remission in 43%, overall response in 79%	[11]						
	Follicular NHL and diffuse large B-cell lymphoma	dsFv IgG4	Calcicheamin	Inotuzumab ozogamicin	Tumor targeting	DNA cleavage	Objective response rate 68% for follicular NHL and 15% for diffuse large B-cell lymphoma	[12]						

Table S1. *Cont*.

TARGET Ag	TARGET TUMOR	Ab FORMAT	FUSED PROTEIN OR CONJUGATED MOIETY	DRUG NAME	Ab PROPERTY/ INTRINSIC ACTIVITY	ADDED PROPERTY	RELEVANT COMMENT	Ref.	a	b	c	d	e	f
CD30	Lymphoma model	scFv plus Fc of human IGg1	IL-2	HRS3scFv-Fc(IL-2)	Tumor targeting	Enhanced immunity	IFN-□ secretion in SCID mice	[13]						
	Lymphoma model	scFv	pRibonuclease	Ber-H2-scFv-hpRNase	Tumor targeting	Protein synthesis inhibition	Anti-tumor activity	[14]						
	Systemic anaplastic large-cell lymphoma	Chimeric (Mouse x Human) IgG1	Monomethyl auristatin E	Brentuximab vedotin	Tumor targeting	Disrupt microtubule polymerization	87% objective response rate and 57% complete remissions BLA submitted to FDA in May 2011	[15]						
CD33	Acute Myeloid Leukemia (AML)	Humanized IgG4	Calicheamicin	Gemtuzumab ozogamicin	Tumor targeting	DNA cleavage	Withdraw from the US market in 2010	[16]						2000
CD64	AML	Humanized scFv	hRNase Angiogenin	Unknown	Tumor targeting	tRNA-specific RNase-mediated protein synthesis inhibition	Prolonged serum half-life	[17]						
Ang1 and 2	Tumor Angiogenesis	Fc fusion	Peptide inhibitor of angiopoietin-1 and -2 receptor, Tie2	AMG 386	Extended *in vivo* half-life	Tumor angiogenesis inhibition	Evidence of antitumor activity	[18]						
ED-A	Renal cell cancer mouse model	Fab diabody	IL-2	F8-(IL-2)	Directed cytokine targeting	Increased leukocyte infiltration	2 out of 7 complete cures, in combination with sunitinib	[19]						
	F9 murine teratocarcinoma	ScFv	IL-12	F8-IL-12	Directed cytokine targeting			[20]						

Table S1. *Cont.*

TARGET Ag	TARGET TUMOR	Ab FORMAT	FUSED PROTEIN OR CONJUGATED MOIETY	DRUG NAME	Ab PROPERTY/ INTRINSIC ACTIVITY	ADDED PROPERTY	RELEVANT COMMENT	Ref.	a	b	c	d	e	f
ED-B	F9 and A431 mouse models of cancer	Human IgG SIP	Porphyrin-based photosensitiser	Unknown	Tumor targeting	Light irradiation-mediated tumor vasculature disruption	NK-mediated long-lasting complete responses	[21]						
	F9 and C51 mouse models of cancer	Human ScFv	GM-CSF	L19-(GM-CSF)	Tumor targeting	$CD8^+$ T-cell involvement	Anti-tumoral and anti-metastatic effect	[22]						
	F9 and C51 mouse models of cancer	Human ScFv	IL-15	L19-(IL15)	Tumor targeting	$CD8^+$ T-cell involvement	Anti-tumoral and anti-metastatic effect	[22]						
	F9 mouse model of cancer	Human ScFv	IL-12	L19-(IL-12)	Tumor targeting	Targeting IL-12 to tumor site	Synergy w/ L19-TNFa	[23]						
	Human tumor xenograft	Humanized IgG1	IL-12	huBC1-IL-12	Incresased binding affinity	Targeting IL-12 to tumor site	Tumor size and metastases reduction	[24]						
EGFR	Human tumor xenograft	C225 Ab-derived scFv	HLA-A2-peptide complexes	scFv- MHC-peptide	Tumor targeting	Ag presentation bypass	CTL-dependent lysis	[25]						
EpCAM	Epithelial carcinomas	Humanized IgG1	IL-2	EMD 273066 (huKS-IL2)	Tumor targeting	ADCC and increased immunological activity	Chills, rigor, anemia as adverse events	[26]						
	Murine Lewis lung cancer	Humanized IgG1	IL-2/IL-12	KS-(IL-12/IL-2)	Tumor targeting	Induction of IFN-g secretion	Similar to immunocytokine combination	[27]						
	Xenograft colon cancer model	Murine heterominibody	mGM-CSF/IL-2	DCH-GM-CSF/IL2	Tumor targeting	T-cell and PBMC mediated cytotoxicity	No GM-CSF/IL2 synergy observed	[28]						

Table S1. *Cont*.

TARGET Ag	TARGET TUMOR	Ab FORMAT	FUSED PROTEIN OR CONJUGATED MOIETY	DRUG NAME	Ab PROPERTY/ INTRINSIC ACTIVITY	ADDED PROPERTY	RELEVANT COMMENT	Ref.	a	b	c	d	e	f
EpCAM (Cont.)	Gastric cancer	Human heterominibody	hGM-CSF/IL-2		Tumor targeting	PBMC mediated cytotoxicity *in vitro* & T-cell redirected lysis		[29]						
	Metastatic melanoma model	C215 Ab-derived Fab	SEA	C215Fab-SEA	Tumor targeting	Superantigen-directed T-cell activation	Synergy observed when administered in combination w/docetaxel or INF-α	[30]						
FAP	FAP-expressing HT1080 cell line	scFv36	41-BBL (CD137L)	scFv36-4-1BBL	Tumor targeting	T-cell co-stimulation	In combination w/bispecific antibodies	[31]						
		ScFv	mGITR Ligand	Anti-FAP-mGITRL	Cancer-associated fibroblast targeting	T-cell activation	Overcome T-reg supression	[32]						
	Tumor xenograft mouse model	scFv	TNF-α	Anti-FAP-TNF dimer	Tumor targeting	TNF-mediated macrophage recruitment into tumor tissue	Combination w/anti-FAP-IL-8 increased antitumoral activity in mouse xenografts models	[33]						
	Tumor xenograft mouse model	scFv	IL-8	Anti-FAP-IL8	Tumor targeting	PMN chemoattraction	Combination w/anti-FAP-TNF increased antitumoral activity in mouse xenografts models	[33]						
G_{D2}	Relapsed/ refractory neuroblastoma	Humanized IgG1	IL-2	Hu 14.18-(IL-2)/ EMD 273063	Tumor targeting	Increased ADCC and immunological activity	Stable disease, 21.7% CR rate in patients with minimal disease	[34]						
		Humanized IgG1	GM-CSF	Hu 14.18-(GM-CSF)	Tumor targeting	Increased PMN-mediated ADCC	Mac-1 dependent ADCC	[35]						

Table S1. *Cont.*

TARGET Ag	TARGET TUMOR	Ab FORMAT	FUSED PROTEIN OR CONJUGATED MOIETY	DRUG NAME	Ab PROPERTY/ INTRINSIC ACTIVITY	ADDED PROPERTY	RELEVANT COMMENT	Ref.	a	b	c	d	e	f
HER2/*neu*	Breast cancer	Humanized IgG1	DM1	Trastuzumab emtansine	Microtubule disrupting agent	Activity on refractory tumor cells	In Phase II, ORR 39.5%	[36]						
	Tumor xenografts mouse model	ScFv-Fc	IL-2	HFI	Tumor targeting	Improved ADCC *in vitro*	Reduction of tumor growth	[37]						
		Human IgG3	IL-2	anti-HER2/*neu* IgG3-(IL-2)	Tumor targeting	Immune stimulation	Tumor growth retardation, protection	[38]						
		Human IgG3	GM-CSF	anti-HER2/*neu* IgG3-(GM-CSF)	Tumor targeting	Enhanced Th1 and Th2 immune response	Tumor growth retardation, protection	[38]						
		Human IgG3	IL-12	anti-HER2/*neu* (IL-12)	Tumor targeting	Involvement of T- and NK cells	Tumor growth retardation, protection	[39]						
		ScFv	TNF	scFv23/TNF	Tumor targeting	Increased apoptosis induction	Induces TNFR expression on tumor cells	[40]						
		ScFv	hRNase	Erbicin-hRNase	Her2 epitope different from trastuzumab	Signalling pathway inhibition	no cardiotoxicity	[41]						
		ScFv-Fc	none	Erbicin-hcAb	Her2 epitope different from trastuzumab		no cardiotoxicity	[41]						
	Human breast tumor cell lines	Human IgG3	C5a	Anti-HER2/*neu* IgG3(C5a)	Specific cytotoxicity	Increased PMN survival and activation	Multiple mechanisms	[42]						
	$HER2^+$ melanoma model	scFv	aGalCer-loaded CD1d	sCD1d-anti-HER2	Tumor targeting	iNKT activation	Reduced metastases and tumor size	[43]						

Table S1. *Cont*.

TARGET Ag	TARGET TUMOR	Ab FORMAT	FUSED PROTEIN OR CONJUGATED MOIETY	DRUG NAME	Ab PROPERTY/ INTRINSIC ACTIVITY	ADDED PROPERTY	RELEVANT COMMENT	Ref.	a	b	c	d	e	f
HLA class II/CD89	Lymphoma	Bispecific scFv	none	FcaRI x HLA class II	Tumor/effector targeting	PMN recruitment	Cytotoxic to resistant primary tumor cells	[44]						
IL-13Ra2	Glioma tumor model	scFv	PE38	anti-IL-13Ra2 (scFv)-PE38	IL-13Ra2 overexpressing-tumors targeting	Transcription inhibition	Specific anti-tumor activity in mouse models	[45]						
Lewis Y	Rat brain tumor model	Chimeric (Mouse x Human) IgG	Doxorubicin	BR96-DOX	Tumor targeting	Standard chemotherapeutic drug delivery	No better than free drug	[46]						
MEL (gp240)	Melanoma	scFv	TNF	scFvMEL/TNF	Tumor targeting	Inhibition of SAPK/JNK pathway	Complete regression in athymic mice	[47]						
MUC-1	Mammary cancer	scFv-Fc of human IgG1	IL-2	C595 scFv-Fc(IL-2)	Tumor targeting	NK activation		[48]						
NCR ligands	Prostate cancer	Human Fc IgG1	NKp30	NKp30-Ig	Binding to NK targets	Macrophage-mediated lysis	Suppression of tumor development	[49]						
OC183B2	Ovarian cancer	Murine scFv	IL-2	IL-2-183B2-scFv	Tumor targeting	IL-2 delivery to tumor site	Biologically active	[50]						
PMSA	Prostate cancer	MoAb	antimicrobial KLA	MLN591-KLA	Tumor targeting	apoptosis induction	Increased cytotoxicity	[51]						
Tenascin-C	Tumoral angiogenesis Glioblastoma xenograft model	Human Ig	IL-2	F16-(IL-2)	Tumor targeting	Leukocyte recruitment into tumors	100% CR in mice	[52]						
	Gliomas and lung tumors	scFv	IL-2	scFv(G11)-(IL-2)	Tumor targeting	IL-2 delivery	Strong staining of human lung tumor sections	[53]						

Table S1. *Cont.*

TARGET Ag	TARGET TUMOR	Ab FORMAT	FUSED PROTEIN OR CONJUGATED MOIETY	DRUG NAME	Ab PROPERTY/ INTRINSIC ACTIVITY	ADDED PROPERTY	RELEVANT COMMENT	Ref.	a	b	c	d	e	f
Tissue factor	Uterine serous papillary carcinoma	Fc fusion	factor VII	hI-conI	Higher antigen affinity	ADCC	Strong staining of human ovarian tumor sections	[54]						
Transferrin Receptor	Several	Chimeric (Mouse x Human) IgG3	Avidin	Anti-hTfR IgG3-Av	Tumor targeting	Increased receptor degradation	Improved with Gambogic acid	[55]						
"TNT"	Solid tumors	Murine IgG	Murine 41-BBL (CD137L)	TNT-3/CD137L	Targeting of necrotic regions	T-cell co-stimulation	T-cell dependent tumor regression	[56]						
VEGF	Angiogenesis	Fc fusion	ECD of VEGFR-1 and -2	Aflibercept	Targeting tumor vasculature	Extended *in vivo* half-life	Modest activity in non-small lung cell carcinoma. Promising results in metastatic colon adenocarcinoma	[57]						

Ag, antigen. Ab, antibody. Ref, reference. a to f refer to development status: a, in vitro. b, in vivo. c, d and e, clinical studies on Phase I, II and III, respectively. f, FDA approved.

References

1. Forsberg, G.; Skartved, N.J.; Wallen-Ohman, M.; Nyhlen, H.C.; Behm, K.; Hedlund, G.; Nederman, T. Naptumomab estafenatox, an engineered antibody-superantigen fusion protein with low toxicity and reduced antigenicity. *J. Immunother.* **2010**, *33*, 492-499.
2. Bauer, S.; Oosterwijk-Wakka, J.C.; Adrian, N.; Oosterwijk, E.; Fischer, E.; Wüest, T.; Stenner, F.; Perani, A.; Cohen, L.; Knuth, A.; Divgi, C.; Juger, D.; Scott, A.M.; Ritter, G.; Old, L.J.; Renner, C. Targeted therapy of renal cell carcinoma: Synergistic activity of cG250-TNF and IFNg. *Int. J. Cancer* **2009**, *125*, 115-123.
3. Ishii, T.; Ishida, T.; Utsunomiya, A.; Inagaki, A.; Yano, H.; Komatsu, H.; Iida, S.; Imada, K.; Uchiyama, T.; Akinaga, S.; Shitara, K.; Ueda, R. Defucosylated Humanized Anti-CCR4 Monoclonal Antibody KW-0761 as a Novel Immunotherapeutic Agent for Adult T-cell Leukemia/Lymphoma. *Clin. Cancer Rese.* **2010**, *16*, 1520-1531.
4. Rider, D.A.; Havenith, C.E.G.; de Ridder, R.; Schuurman, J.; Favre, C.; Cooper, J.C.; Walker, S.; Baadsgaard, O.; Marschner, S.; vandeWinkel, J.G.J.; Cambier, J.; Parren, P.W.H.I.; Alexander, D.R. A Human CD4 Monoclonal Antibody for the Treatment of T-Cell Lymphoma Combines Inhibition of T-Cell Signaling by a Dual Mechanism with Potent Fc-Dependent Effector Activity. *Cancer Res.* **2007**, *67*, 9945-9953.

5. Link, B.K.; Martin, P.; Kaminski, M.S.; Goldsmith, S.J.; Coleman, M.; Leonard, J.P. Cyclophosphamide, Vincristine, and Prednisone Followed by Tositumomab and Iodine-131 Tositumomab in Patients With Untreated Low-Grade Follicular Lymphoma: Eight-Year Follow-Up of a Multicenter Phase II Study. *J. Clin. Oncol.* **2010**, *28*, 3035-3041.
6. Hainsworth, J.; Spigel, D.; Markus, T.; Shipley, D.; Thompson, D.; Rotman, R.; Dannaher, C.; Greco, F.A. Rituximab plus Short-Duration Chemotherapy Followed by Yttrium-90 Ibritumomab Tiuxetan as First-Line Treatment for Patients with Follicular Non-Hodgkin's Lymphoma: A Phase II Trial of the Sarah Cannon Oncology Research Consortium. *Clin. Lymphoma Myeloma Leukemia* **2009**, *9*, 223-228.
7. Liu, R.; Jiang, W.; Yang, M.; Guo, H.; Zhang, Y.; Wang, J.; Zhu, H.; Shi, R.; Fan, D.; Yang, C.; Zhu, Z.; Xie, Y.; Xiong, D. Efficient inhibition of human B-cell lymphoma in SCID mice by synergistic antitumor effect of human 4-1BB Ligand/Anti-CD20 fusion proteins and Anti-CD3/Anti-CD20 diabodies. *J. Immunother.* **2010**, *33*, 500-509.
8. Gillies, S.D.; Lan, Y.; Williams, S.; Carr, F.; Forman, S.; Raubitschek, A.; Lo, K.-M. An anti-CD20-IL-2 immunocytokine is highly efficacious in a SCID mouse model of established human B lymphoma. *Blood* **2005**, *105*, 3972-3978.
9. Xuan, C.; Steward, K.K.; Timmerman, J.M.; Morrison, S.L. Targeted delivery of interferon-alpha via fusion to anti-CD20 results in potent antitumor activity against B-cell lymphoma. *Blood* **2010**, *115*, 2864-2871.
10. Imai, M.; Ohta, R.; Varela, J.C.; Song, H.; Tomlinson, S. Enhancement of antibody-dependent mechanisms of tumor cell lysis by a targeted activator of complement. *Cancer Res.* **2007**, *67*, 9535-9541.
11. Kreitman, R.J.; Tallman, M.S.; Coutre, S.E.; Robak, T.; Wilson, W.H.; Stetler-Stevenson, M.; Noel, P.; FitzGerald, D.J.; McDevitt, J.T.; Pastan, I. Phase I trial of recombinant immunotoxin CAT-8015 (HA22) in multiply relapsed hairy cell leukemia. *J. Clin. Oncology ASCO Meeting Abstracts* **2010**, *28*, 6523.
12. Advani, A.; Coiffier, B.; Czuczman, M.S.; Dreyling, M.; Foran, J.; Gine, E.; Gisselbrecht, C.; Ketterer, N.; Nasta, S.; Rohatiner, A.; Schmidt-Wolf, I.G.H.; Schuler, M.; Sierra, J.; Smith, M.R.; Verhoef, G.; Winter, J.N.; Boni, J.; Vandendries, E.; Shapiro, M.; Fayad, L. Safety, Pharmacokinetics, and Preliminary Clinical Activity of Inotuzumab Ozogamicin, a Novel Immunoconjugate for the Treatment of B-Cell Non-Hodgkin's Lymphoma: Results of a Phase I Study. *J. Clin. Oncol.* **2010**, *28*, 2085-2093.
13. Hirsch, B.; Brauer, J.; Fischdick, M.; Loddenkemper, C.; Bulfone-Paus, S.; Stein, H.; Dürkop, H. Anti-CD30 human IL-2 fusion proteins display strong and specific cytotoxicity *In Vivo*. *Curr. Drug Targets* **2009**, *10*, 110-117.
14. Braschoss, S.; Hirsch, B.; Dübel, S.; Stein, H.; Dürkop, H. New anti-CD30 human pancreatic ribonuclease-based immunotoxin reveals strong and specific cytotoxicity in vivo. *Leukemia Lymphoma* **2007**, *48*, 1179-1186.
15. Shustov, A.R.; Advani, R.; Brice, P.; Bartlett, N.L.; Rosenblatt, J.D.; Illidge, T.; Matous, J.; Ramchandren, R.; Fanale, M.A.; Connors, J.M.; Yang, Y.; Sievers, E.L.; Kennedy, D.A.; Pro, B. In *Complete Remissions with Brentuximab Vedotin (SGN-35) in Patients with Relapsed or Refractory Systemic Anaplastic Large Cell Lymphoma*, ASH Annual Meeting Abstracts, 2010; p. 961.
16. Lowenberg, B.; Beck, J.; Graux, C.; van Putten, W.; Schouten, H.C.; Verdonck, L.F.; Ferrant, A.; Sonneveld, P.; Jongen-Lavrencic, M.; von Lilienfeld-Toal, M.; Biemond, B.J.; Vellenga, E.; Breems, D.; de Muijnck, H.; Schaafsma, R.; Verhoef, G.; Dohner, H.; Gratwohl, A.; Pabst, T.; Ossenkoppele, G.J.; Maertens, J. Gemtuzumab ozogamicin as postremission treatment in AML at 60 years of age or more: results of a multicenter phase 3 study. *Blood* **2010**, *115*, 2586-2591.
17. Hetzel, C.; Bachran, C.; Fischer, R.; Fuchs, H.; Barth, S.; Stöcker, M. Small Cleavable Adapters Enhance the Specific Cytotoxicity of a Humanized Immunotoxin Directed Against CD64-positive Cells. *J. Immunother.* **2008**, *31*, 370-376

18. Karlan, B.Y.; Oza, A.M.; Hansen, V.L.; Richardson, G.E.; Provencher, D.M.; Ghatage, P.; Tassoudji, M.; Stepan, D.E.; Weinreich, D.M.; I.B., V. Randomized, double-blind, placebo-controlled phase II study of AMG 386 combined with weekly paclitaxel in patients (pts) with recurrent ovarian carcinoma. *J. of Clin. Oncology. ASCO Annual Meeting Proceedings (Post-Meeting Edition).* **2010**, *28*, 5000.
19. Frey, K.; Schliemann, C.; Schwager, K.; Giavazzi, R.; Johannsen, M.; Neri, D. The immunocytokine F8-IL2 improves the therapeutic performance of sunitinib in a mouse model of renal cell carcinoma. *J. Urol.* **2010**, *184*, 2540-2548.
20. Sommavilla, R.; Pasche, N.; Trachsel, E.; Giovannoni, L.; Roesli, C.; Villa, A.; Neri, D.; Kaspar, M. Expression, engineering and characterization of the tumor-targeting heterodimeric immunocytokine F8-IL12. *Protein Engineering Design Selection* **2010**, *23*, 653-661.
21. Palumbo, A.; Hauler, F.; Dziunycz, P.; Schwager, K.; Soltermann, A.; Pretto, F.; Alonso, C.; Hofbauer, G.F.; Boyle, R.W.; Neri, D. A chemically modified antibody mediates complete eradication of tumours by selective disruption of tumour blood vessels. *Br. J. Cancer* **2011**, *104*, 1106-1115.
22. Kaspar, M.; Trachsel, E.; Neri, D. The Antibody-Mediated Targeted Delivery of Interleukin-15 and GM-CSF to the Tumor Neovasculature Inhibits Tumor Growth and Metastasis. *Cancer Res.* **2007**, *67*, 4940-4948.
23. Halin, C.; Gafner, V.; Villani, M.E.; Borsi, L.; Berndt, A.; Kosmehl, H.; Zardi, L.; Neri, D. Synergistic Therapeutic Effects of a Tumor Targeting Antibody Fragment, Fused to Interleukin 12 and to Tumor Necrosis Factor alpha. *Cancer Res.* **2003**, *63*, 3202-3210.
24. Lo, K.M.; Lan, Y.; Lauder, S.; Zhang, J.; Brunkhorst, B.; Qin, G.; Verma, R.; Courtenay-Luck, N.; Gillies, S.D. huBC1-IL12, an immunocytokine which targets EDB-containing oncofetal fibronectin in tumors and tumor vasculature, shows potent anti-tumor activity in human tumor models. *Cancer Immunol. Immunother.* **2007**, *56*, 447-457.
25. Novak, H.; Noy, R.; Oved, K.; Segal, D.; Wels, W.S.; Reiter, Y. Selective antibody-mediated targeting of class I MHC to EGFR-expressing tumor cells induces potent antitumor CTL activity *in vitro* and *in vivo*. *Int. J. Cancer* **2007**, *120*, 329-336.
26. Ko, Y.J.; Bubley, G.J.; Weber, R.; Redfern, C.; Gold, D.P.; Finke, L.; Kovar, A.; Dahl, T.; S.D., G. Safety, pharmacokinetics, and biological pharmacodynamics of the immunocytokine EMD 273066 (huKS-IL2): results of a phase I trial in patients with prostate cancer. *J. Immunother.* **2004**, *27*, 232-239.
27. Gillies, S.D.; Lo, K.-M.; Burger, C.; Lan, Y.; Dahl, T.; Wong, W.-K. Improved Circulating Half-Life and Efficacy of an Antibody-Interleukin 2 Immunocytokine Based on Reduced Intracellular Proteolysis. *Clin. Cancer Res.* **2002**, *8*, 210-216.
28. Schanzer, J.M.; Fichtner, I.; Baeuerle, P.A.; Kufer, P. Antitumor activity of a dual cytokine/single-chain antibody fusion protein for simultaneous delivery of GM-CSF and IL-2 to Ep-CAM expressing tumor cells. *J. Immunother.* **2006**, *29*, 477-488.
29. Schanzer, J.M.; Baeuerle, P.A.; Dreier, T.; Peter Kufer, P. A human cytokine/single-chain antibody fusion protein for simultaneous delivery of GM-CSF and IL-2 to Ep-CAM overexpressing tumor cells. *Cancer Immunity* **2006**, *6*, 4.
30. Sundstedt, A.; Celander, M.; Ohman, M.W.; Forsberg, G.; Hedlund, G. Immunotherapy with tumor-targeted superantigens (TTS) in combination with docetaxel results in synergistic anti-tumor effects. *International Immunopharmacology* **2009**, *9*, 1063-1070.
31. Muller, D.; Frey, K.; Kontermann, R.E. A novel antibody-4-1BBL fusion protein for targeted costimulation in cancer immunotherapy. *J. Immunother.* **2008**, *31*, 714-722.

32. Burckhart, T.; Thiel, M.; Nishikawa, H.; Wüest, T.; Muller, D.; Zippelius, A.; Ritter, G.; Old, L.; Shiku, H.; Renner, C. Tumor-specific crosslinking of GITR as costimulation for immunotherapy. *J. Immunother.* **2010**, *33*, 925-934.
33. Bauer, S.; Adrian, N.; Siebenborn, U.; Fadle, N.; Plesko, M.; Fischer, E.; Wüest, T.; Stenner, F.; Mertens, J.C.; Knuth, A.; Ritter, G.; Old, L.J.; Renner, C. Sequential cancer immunotherapy: Targeted activity of dimeric TNF and IL-8. *Cancer Immunity* **2009**, *9*, 2.
34. Shusterman, S.; London, W.B.; Gillies, S.D.; Hank, J.A.; Voss, S.D.; Seeger, R.C.; Reynolds, C.P.; Kimball, J.; Albertini, M.R.; Wagner, B.; Gan, J.; Eickhoff, J.; DeSantes, K.B.; Cohn, S.L.; Hecht, T.; Gadbaw, B.; Reisfeld, R.A.; Maris, J.M.; Sondel, P.M. Antitumor Activity of Hu14.18-IL2 in Patients With Relapsed/Refractory Neuroblastoma: A Children's Oncology Group (COG) Phase II Study. *J. Clin. Oncol.* **2010**, *28*, 4969-4975.
35. Metelitsa, L.S.; Gillies, S.D.; Super, M.; Shimada, H.; Reynolds, C.P.; Seeger, R.C. Antidisialoganglioside/granulocyte macrophage colony-stimulating factor fusion protein facilitates neutrophil antibody-dependent cellular cytotoxicity and depends on Fc(gamma)RII (CD32) and Mac-1 (CD11b/CD18) for enhanced effector cell adhesion and azurophil granule exocytosis. *Blood* **2002**, *99*, 4166-4173.
36. Burris III, H.A. Trastuzumab emtansine: a novel antibody-drug conjugate for HER2-positive breast cancer. *Expert Opin. Biol. Ther.* **2011**, *11*, 807-819.
37. Shi, M.; Zhang, L.; Gu, H.-t.; Jiang, F.-q.; Qian, L.; Yu, M.; Chen, G.-j.; Luo, Q.; Shen, B.-f.; Guo, N. Efficient growth inhibition of ErbB2-overexpressing tumor cells by anti-ErbB2 ScFv-Fc-IL-2 fusion protein in vitro and in vivo. *Acta Pharmacologica Sinica* **2007**, *28*, 1611-1620.
38. Dela Cruz, J.S.; Trinh, K.R.; Chen, H.W.; Ribas, A.; Morrison, S.L.; Penichet, M.L. Anti-HER2/neu IgG3-(IL-2) and anti-HER2/neu IgG3-(GM-CSF) promote HER2/neu processing and presentation by dendritic cells: Implications in immunotherapy and vaccination strategies. *Mol. Immunol.* **2006**, *43*, 667-676.
39. Helguera, G.; Dela Cruz, J.S.; Lowe, C.; Ng, P.P.; Trinh, R.; Morrison, S.L.; Penichet, M.L. Vaccination with novel combinations of anti-HER2/neu cytokines fusion proteins and soluble protein antigen elicits a protective immune response against HER2/neu expressing tumors. *Vaccine* **2006**, *24*, 304-316.
40. Lyu, M.-A.; Rosenblum, M.G. The immunocytokine scFv23/TNF sensitizes HER-2/neu‚Äìoverexpressing SKBR-3 cells to tumor necrosis factor (TNF) via up-regulation of TNF receptor-1. *Mol. Cancer Ther.* **2005**, *4*, 1205-1213.
41. Gelardi, T.; Damiano, V.; Rosa, R.; Bianco, R.; Cozzolino, R.; Tortora, G.; Laccetti, P.; D'Alessio, G.; De Lorenzo, C. Two novel human anti-ErbB2 immunoagents are active on trastuzumab-resistant tumours. *Br. J. Cancer* **2010**, *102*, 513-519.
42. Fuenmayor, J.; Perez-Vazquez, K.; Perez-Witzke, D.; Penichet, M.L.; Montano, R.F. Decreased survival of human breast cancer cells expressing HER2/neu on in vitro incubation with an anti-HER2/neu antibody fused to C5a or C5a desArg. *Mol. Cancer Ther.* **2010**, *9*, 2175-2185.
43. Stirnemann, K.; Romero, J.F.; Baldi, L.; Robert, B.; Cesson, V.; Besra, G.S.; Zauderer, M.; Wurm, F.; Corradin, G.; Mach, J.P.; MacDonald, H.R.; Donda, A. Sustained activation and tumor targeting of NKT cells using a CD1d-anti-HER2-scFv fusion protein induce antitumor effects in mice. *J. Clin. Invest.* **2008**, *118*, 994-1005.
44. Guettinger, Y.; Barbin, K.; Peipp, M.; Bruenke, J.; Dechant, M.; Horner, H.; Thierschmidt, D.; Valerius, T.; Repp, R.; Fey, G.H.; Stockmeyer, B. A recombinant bispecific single-chain fragment variable specific for HLA class II and FcγRI (CD89) recruits polymorphonuclear neutrophils for efficient lysis of malignant B lymphoid cells. *J. Immunol.* **2010**, *184*, 1210-1217.

45. Kioi, M.; Seetharam, S.; Puri, R.K. Targeting IL-13RA2-positive cancer with a novel recombinant immunotoxin composed of a single-chain antibody and mutated Pseudomonas exotoxin. *Mol. Cancer Ther.* **2008**, *7*, 1579-1587.
46. Muldoon, L.L.; Neuwelt, E.A. BR96–DOX Immunoconjugate Targeting of Chemotherapy in Brain Tumor Models. *J. Neuro-Oncol.* **2003**, *65*, 49-62.
47. Liu, Y.; Zhang, W.; Cheung, L.H.; Niu, T.; Wu, Q.; Li, C.; Van Pelt, C.S.; Rosenblum, M.G. The antimelanoma immunocytokine scFvMEL/TNF shows reduced toxicity and potent antitumor activity against human tumor xenografts. *Neoplasia* **2006**, *8*, 384-393.
48. Heuser, C.; Ganser, M.; Hombach, A.; Brand, H.; Denton, G.; Hanisch, F.G.; Abken, H. An anti-MUC1-antibody-interleukin-2 fusion protein that activates resting NK cells to lysis of MUC1-positive tumour cells. *Br. J. Cancer* **2003**, *89*, 1130-1139.
49. Arnon, T.I.; Markel, G.; Bar-Ilan, A.; Hanna, J.; Fima, E.; Benchetrit, F.; Galili, R.; Cerwenka, A.; Benharroch, D.; Sion-Vardy, N.; Porgador, A.; Mandelboim, O. Harnessing soluble NK cell killer receptors for the generation of novel cancer immune therapy. *PLoS ONE* **2008**, *3*.
50. Zhang, X.; Feng, J.; Ye, X.; Yao, Y.; Zhou, P.; Chen, X. Development of an immunocytokine, IL-2-183B2scFv, for targeted immunotherapy of ovarian cancer. *Gynecologic Oncol.* **2006**, *103*, 848-852.
51. Rege, K.; Patel, S.J.; Megeed, Z.; Yarmush, M.L. Amphipathic peptide-based fusion peptides and immunoconjugates for the targeted ablation of prostate cancer cells. *Cancer Res.* **2007**, *67*, 6368-6375.
52. Pedretti, M.; Verpelli, C.; Marlind, J.; Bertani, G.; Sala, C.; Neri, D.; Bello, L. Combination of temozolomide with immunocytokine F16-IL2 for the treatment of glioblastoma. *Br. J Cancer* **2010**, *103*, 827-836.
53. Silacci, M.; Brack, S.S.; Späth, N.; Buck, A.; Hillinger, S.; Arni, S.; Weder, W.; Zardi, L.; Neri, D. Human monoclonal antibodies to domain C of tenascin-C selectively target solid tumors in vivo. *Protein Engineering, Design and Selection* **2006**, *19*, 471-478.
54. Cocco, E.; Hu, Z.; Richter, C.E.; Bellone, S.; Casagrande, F.; Bellone, M.; Todeschini, P.; Krikun, G.; Silasi, D.A.; Azodi, M.; Schwartz, P.E.; Rutherford, T.J.; Buza, N.; Pecorelli, S.; Lockwood, C.J.; Santin, A.D. HI-con1, a factor VII-IgGFc chimeric protein targeting tissue factor for immunotherapy of uterine serous papillary carcinoma. *Br. J. Cancer* **2010**, *103*, 812-819.
55. Ortiz-Sanchez, E.; Daniels, T.R.; Helguera, G.; Martinez-Maza, O.; Bonavida, B.; Penichet, M.L. Enhanced cytotoxicity of an anti-transferrin receptor IgG3-avidin fusion protein in combination with gambogic acid against human malignant hematopoietic cells: Functional relevance of iron, the receptor, and reactive oxygen species. *Leukemia* **2009**, *23*, 59-70.
56. Zhang, N.; Sadun, R.E.; Arias, R.S.; Flanagan, M.L.; Sachsman, S.M.; Nien, Y.C.; Khawli, L.A.; Hu, P.; Epstein, A.L. Targeted and untargeted CD137L fusion proteins for the immunotherapy of experimental solid tumors. *Clin. Cancer Res.* **2007**, *13*, 2758-2767.
57. Tarhini, A.A.; Christensen, S.; Frankel, P.; Margolin, K.; Ruel, C.; Shipe-Spotloe, J.; DeMark, M.; Kirkwood, J.M. Phase II study of aflibercept (VEGF trap) in recurrent inoperable stage III or stage IV melanoma of cutaneous or ocular origin. *ASCO Meeting Abstracts* **2009**, *27*, 9028.

© 2011 by the authors; licensee MDPI, Basel, Switzerland. This article is an open-access article distributed under the terms and conditions of the Creative Commons Attribution license (http://creativecommons.org/licenses/by/3.0/).
